# Full stage networks with auxiliary focal loss and multi-attention module for submarine garbage object detection

**DOI:** 10.1038/s41598-023-42896-3

**Published:** 2023-09-26

**Authors:** Hui Zheng, Xinwei Guo, Guihai Guo, Yizhi Cao, Xinglei Hu, Pujie Yue

**Affiliations:** 1 Ural Institute, North China University of Water Resources and Electric Power, Zhengzhou, 450045 China; 2 School of Mechanical Engineering, Shanghai Jiao Tong University, Shanghai, 200240 China; 3 Shanghai Marine Diesel Engine Research Institute, Shanghai, 201100 China; 4 China Energy Digital Technology Group Co, Ltd, Beijing, 100022 China

**Keywords:** Computer science, Environmental sciences, Ocean sciences

## Abstract

Submarine garbage is constantly destroying the marine ecological environment and polluting the ocean. It is critical to use detection methods to quickly locate and identify submarine garbage. The background of submarine garbage images is much more complex than that of natural scene images, with object deformation and missing contours putting higher demands on the detection network. To solve the problem of low accuracy under complex backgrounds, full stage networks with auxiliary focal loss and multi-attention module are proposed for submarine garbage object detection based on YOLO. To maximize the gradient combination, a hierarchical fusion feature mechanism and a segmentation and merging strategy are used in this paper to optimize the difference in gradient combination to obtain full-stage features. Then the criss-cross attention module is used to precisely extract multi-scale features of small object dense regions while removing noise information from complex backgrounds. Finally, the auxiliary focal loss function addresses the issue of unbalanced positive and negative samples, focusing on the learning of difficult samples while improving overall detection precision. Based on comparative experiments and ablation experiments, the FSA networks achieved state-of-the-art performance, and is applicable to the real-time object detection of submarine garbage in complex backgrounds.

## Introduction

Submarine garbage is becoming an increasingly serious issue in the marine ecological environment. Due to poor management, a large amount of garbage generated using artificial products would enter the marine environment, causing serious pollution of the ocean. Wood, fishing nets, glass, metals, plastics, and other durable and corrosion-resistant materials may be found in garbage, and once in the ocean, they become persistent pollutants. As a result, controlling and managing submarine garbage pollution is critical^1–4^. On technological level, the detection method of submarine garbage, that is, rapidly locating and identifying submarine garbage using detection, obtaining basic information on the distribution and quantity of submarine garbage pollution, and formulating control policies, is a critical link in promoting submarine garbage pollution cleanup and recycling.

The majority of the studies use remote sensing technology to detect and classify marine floating plastic wastes; however, few scholars have studied submarine garbage, and many types of garbage greatly increase the difficulty of object detection. Xu^5^ employed YOLOv3 to detect fish in underwater environments for waterpower applications, and achieved a mean average precision (mAP) value of 54.92%. Asyraf^6^ conducted a study on the efficiency of the YOLOv3 detector in detecting underwater life on two open-source datasets, and the results indicate that the YOLOv3 detector is capable of detecting underwater objects with high accuracy, with mAP scores ranging from 74.88 to 97.56%. Rosli^7^ used YOLOv4 to detect underwater animals and the training results showed a mAP of 97.86%. Chen^8^ used YOLOv4 to detect 4757 images with 4 categories on the URPC dataset, and the results showed a mAP of 73.48%. Zhang^9^ trained and tested the URPC dataset with a mAP of 81.01%. Gašparović^10^ improved YOLOv4 and achieved better detection results in underwater pipeline object detection with 94.21% mAP.

Object detection technology is of great significance as the basis of more complex and higher-level visual tasks such as pattern recognition, object tracking, event detection, and activity recognition. Currently, deep learning-based object detection algorithms are divided into two main categories: one-stage object detection and two-stage object detection.

The most classical two-stage algorithm is the R-CNN^11^ (Region-based Convolutional Neural Networks) proposed by Grishick^12^ based on the AlexNet architecture combining region proposal with CNN, but this detector is more time consuming. He proposed the detector of SPPNet (Spatial Pyramid Pooling Networks)^13^, which solved the time-consuming problem of R-CNN. Grishick^14^ proposed Fast R-CNN detector based on R-CNN and SPPNet, which improved the mAP (mean Average Precision) to 70.0% and reduced the elapsed time. Ren^15^ proposed the Faster R-CNN detector based on the RPN (Region Proposal Networks) which unifies the generation of candidate regions, feature extraction, confirmation of candidate objects, and border coordinate regression into the same network framework. Dai^16^ proposed a region-based detector R-FCN (Region-based Fully Convolutional Networks) based on FCN^17^ (Fully Convolutional Networks) to solve the contradiction between the location insensitivity of classification networks and the location sensitivity of detection networks. Lin^18^ proposed the FPN (Features Pyramid Networks) detector based on Faster R-CNN, which has better detection advantages for small objects and objects with large-scale variations. He^19^ introduced ROI (Region of Interesting) into Faster R-CNN and proposed Mask R-CNN to achieve fast detection and instance segmentation of objects. Cai^20^ proposed a cascade multi-stage network architecture Cascade R-CNN to solve the problem of IoU (Intersect over Union) threshold selection in object detection. Hu^21^ proposed the RelationNet detector by utilizing the interrelationship between objects to optimize the detection effect. Zhang^22^ put forward the RefineDet detector based on SSD^23^ (Single Shot Multibox Detector), adopted the idea of one-stage and two-stage, and integrated SSD, RPN, and FPN algorithms, which can improve the detection effect.

OverFeat^24^ is an early classic one-stage object detection algorithm based on AlexNet, which implements a trinity network framework of recognition, localization, and detection. Redmon^25^ proposed the YOLO algorithm, which takes the object detection as a regression problem, the object position and category information can be output by detecting the image only one time. Ross^26^ proposed RetinaNet based on the ResNet^27^ structure using FPN to compensate for the accuracy discrepancy caused by the one-stage category imbalance. Duan^28^ proposed CenterNet which transforms the detection object bounding box into the detection object centroid, avoiding post-processing by non-maximum suppression and eliminating the need for border regression. Tan^29^ used EfficientNet as backbone and scaled the model using bidirectional feature pyramid network and multiscale features, enhancing advanced feature fusion with better efficiency, accuracy, and smaller size.

Joseph^30^ proposed YOLOv2 based on YOLO, which trains the object detector on both detection and classification datasets, using the data from the detection dataset to learn the exact location of the object and the data from the classification dataset to increase the number of categories for classification. Among the YOLO series of object detection models, YOLOv3^31^ is a classic one-stage model, which is divided into four parts: input, backbone, neck, and prediction. YOLOv4^32^ has made many innovations based on YOLOv3. YOLOv5^33^ mainly calculates the scaling ratio of the original image size and the input size and obtains the scaled image size, and the main difference from YOLOv4 are mosaic data enhancement is adopted at the input, and CSPDarknet^34^ in backbone, mish activation function^35^, drop block, etc. The Neck adopts the structure of SPP^11^ and FPN^18^ with PAN (Path Aggregation Network)^36^, CIoU (Complete IoUnion) ^37^ loss, and DIoU (Distance-IoU) ^38^ NMS(Non-Maximum Suppression) are used in the output. YOLOX^39^combines the best advances in the field of object detection with YOLO, such as decoupling headers, data broadening, label assignment, and anchor-free module, to achieve a significant performance improvement. YOLOv7^40^ is a combination of a collection of existing tricks as well as modular re-referencing and dynamic label assignment strategies, ultimately outperforming the vast majority object detectors in speed and accuracy in the 5 FPS to 160 FPS range. YOLOv8^41^ is a SOTA model that builds on the success of previous YOLO versions and introduces new features and improvements to further enhance performance and flexibility.

PicoDet^42^ is a compact object detector that employs attention processes and multi-scale feature pyramids to enhance detection through one-stage network construction. By aligning task relevance, task-aligned one-stage object detection (TOOD)^43^ addresses the issue of inconsistent categorization and localization predictions in detection tasks and delivers accurate and effective detection. RTMDet^44^is a more recent industrial detector that combines the most recent performance in real-time instance segmentation and rotating object recognition with the best parametric accuracy between tiny, small, medium, large, and oversized model sizes for diverse application scenarios. PP-YOLOE^45^ is an object detection model that improves on the YOLOv3 algorithm with a redesigned network structure and a more efficient convolution operation. This enables PP YOLO to process images in real time while maintaining high detection accuracy.

The YOLO-based network model achieves a balance between detection speed and accuracy and is the most popular use of the one-stage object detection approaches. However, due to the situations of blurred submarine garbage images, incomplete object contours, and deformation of objects captured underwater, object detection of submarine garbage is more challenging. In response to the above situation, this paper identify and detect 15 types of submarine garbage, and proposed a full stage shortcut convolutional neural networks with auxiliary focal loss and multi-attention module for submarine garbage object detection based on the YOLO method, adopting hierarchical fusion feature mechanism alleviates the drawbacks caused by using explicit feature map replication for cascading, adding criss-cross attention module and fusing with full stage cross features can obtain dense features that focus more on small objects, using auxiliary head and weighted focal loss to solve the problem of unbalanced positive and negative samples, solving the problem of difficult extraction of submarine garbage objects in complex backgrounds, and boosting detection accuracy overall, and enriching the identification types of submarine garbage, and providing more reference information for pollution cleaning and recycling of submarine garbage.

## Results

### Experimental environment and parameters

The software environment and hardware parameters used in this paper are shown in Table 1. The hyperparameter of experiments in training FSA networks is shown in Table 2.

**Table 1:** Software and hardware configuration of the experimental environment.

Platform	Configuration
Operating system	Windows 10 OS (64 GB RAM)
CPU model	Intel i7-11,700 CPU
GPU model	NVIDIA RTX A1000 (16G)
Integrated development environment	PyCharm
Scripting language	Python3.9
GPU accelerator	CUDA11.7
Neural network accelerator	cuDNN8.4

**Table 2:** FSA networks experimental training parameters.

Hyperparameter	Configuration
Neural network optimizer	SGD
Training epochs	300
Batch size	16
Weight decay	0.0005
Warmup epochs	3
Initial learning rate	0.01
Final learning rate	0.002
Momentum	0.937

This paper mainly adopts mAP50:95(mAP) as the model evaluation index of performance.

### Baseline experiments

In order to verify the effectiveness of the proposed FSA networks model, ablation experiments are conducted to evaluate the effect of different modules on the performance of the object detection algorithm under the same experimental conditions. Before determining the baseline model, comparison experiments are conducted between YOLOv5 and YOLOv7 series models.

From Table 3, it can be seen that layers, parameters, GFLOPS, and the mAP of the YOLOv5 series all increase with the increase of model size. The mAP reaches its maximum at YOLOv5x. Layers, parameters, and GFLOPS of the YOLOv7 series all increase with the increase in model size, and the mAP reach maximum at YOLOv7-w6. Therefore, in the ablation experiment, YOLOv7 was selected as the baseline model.

**Table 3:** Baseline experiment.

Model	Layers	Params (M)	GFLOPS	mAP (%)
YOLOv7	415	37.23	105	51.5
YOLOv7-x	467	70.91	189.2	52.1
YOLOv7-w6	477	81.19	103	52.1
YOLOv7-e6	645	110.65	144.9	49.8
YOLOv7-d6	733	153.21	199	48.6
YOLOv7-e6e	1063	165.16	227.2	47.7
YOLOv5-s	262	9.13	24.1	48.4
YOLOv5-m	339	25.07	64.4	48.8
YOLOv5-l	416	53.17	135.3	49.5
YOLOv5-x	493	97.21	247	50.90

### Ablation experiments on FSA networks

On COCO datasets, the various YOLO family improvement methods currently perform significantly better, but the extension to bespoke datasets has not yet been thoroughly demonstrated.

In this paper, an FSA network is designed using a highly reused FFS module with highly used features in the backbone and an efficient group convolutional SPPCSPC module in the neck part. Additionally, the criss-cross attention mechanism is connected to the FFS module in the head and combined the features in the backbone, and the object detection task is completed using lead head and auxiliary head. In this section, ablation experiments are conducted to verify the effectiveness of the FSA network and to compare it with the current leading detectors. The results show that the FSA network proposed in this paper achieves state of the art on the submarine garbage dataset.

As can be seen from Table 4, model A uses a combination of HS and FS to extract features using standard convolution with 464 layers, 121.22 M parameters, and an mAP of 52.5%, which demonstrates the ability of the HS and FS modules to extract features while improving accuracy and reducing the number of parameters compared to the YOLOV7 series models. The backbone and head backbone architectures of model B both use FS modules, which have a slight increase in the number of parameters and GFLOPS and a 0.2% increase in mAP compared to HS modules. This is mainly because the FS module retains the detailed features of each layer in the module. In order to further reduce the complexity of the model and the number of parameters, model C uses depthwise separable convolution in the FS module, so that each convolution kernel operates on only one channel and does not change the number of channels, but some channel information is lost. therefore, in the experiment, the kernel size is increased to expand the feature extraction The results show that the FS extracted high-density feature map after depthwise separable convolution has the same accuracy as model A.

**Table 4:** Ablation experiments of parameters with different module.

Model	Backbone + head	Convolution	Layers	Parameters(M)	GFLOPS	mAP (%)
A	HS+FS	Standard convolution	464	121.22	142.1	52.5
B	FS+FS	Standard convolution	464	(+ 9.08) 130.3	152.1	(+ 0.2) 52.7
C	FS+FS	Depthwise separable convolution	464	(− 62.12) 59.1	80.5	52.5
D	FSS+FSS	Depthwise separable convolution	464	(− 62.12) 59.1	80.5	(+ 1.6) 54.1
E	D + CCA	Depthwise separable convolution	490	(− 60.9) 60.32	81.2	(+ 2.5) 55.0
F	E + Auxhead	Depthwise separable convolution	523	(− 59.74) 61.48	84.2	(+ 3.0) 55.5

The FSS module in model D is the final structure adopted in this paper. Compared with the FS module, the FSS module, with the addition of shortcut connections, is similar to the residual structure of ResNet. Moreover, the feature maps of layers P3–P6 in backbone are passed to layers P3–P6 in head, which make up for the lack of information in depthwise separable convolution, and the mAP has a more obvious improvement (+ 1.6%). Model E and model F were trained by adding criss-cross attention mechanism and auxiliary head in turn, and the mAP was improved by 2.5% and 3.0%, respectively, compared with model A. Therefore, the FSA network proposed in this paper has a very significant detection effect.

**Figure 1:**
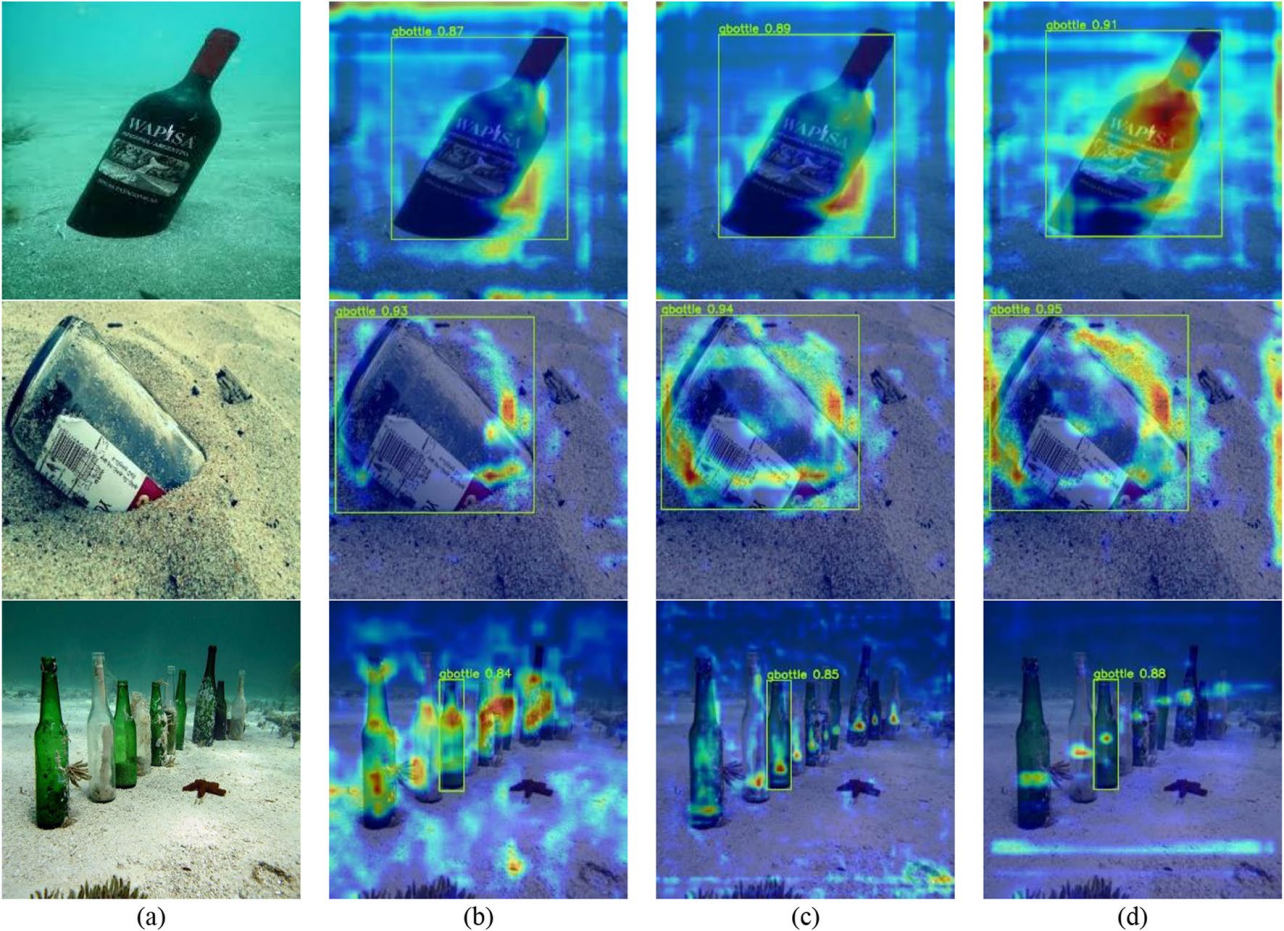
Performance of different feature layers of FSA networks. (**a**) Original image; (**b**) Heatmap of backbone output; (**c**) Heatmap of SPPCSPC output; (**d**) Heatmap of head output.

Figure 1 displays the heat map and detection accuracy at the head, SPPCSPC, and backbone outputs of FSA networks. The benefit of the attention mechanism grows enormously as the network’s depth increases, and accuracy likewise rises.

### Comparisons with state-of-the-art methods

To validate the effectiveness of FSA networks, this paper compares some state-of-the-art methods on the submarine garbage dataset. The models involved in the comparison are two-stage detector and one-stage detector (containing anchor free detectors, such as TOOD, YOLOX). The compared algorithms are trained based on PaddleDetection and MMDetection with epochs set to 100 for two-stage detector and 300 for one-stage detector, and the rest of parameters remain unchanged. The results show that the FSA model achieves 55.5% mAP, which is more accurate than many state-of-the-art methods, and the comparison results are shown in Table 5.

**Table 5:** Comparisons with state-of-the-art methods on submarine garbage test set.

Method	Backbone	Mask	Can	Cellphone	Electronics	Gbottle	Glove	Metal	Misc	Net	Pbag	Pbottle	Plastic	Rod	Sunglasses	Tire	mAP (%)
Two-stage																	
Cascade RCNN^20^	ResNet101	48.3	25.9	79.9	42.5	41.9	59.1	22.5	34.2	60.4	77.5	41.4	25.6	11.9	50.0	29.2	43.4
Faster RCNN^15^	ResNet50	63.2	23.3	74.3	36.5	33.4	76.6	10.2	33.0	53.2	73.3	38.8	16.7	12.2	6.1	28.1	38.6
Faster RCNN^15^	ResNet101	33.4	30.3	77.8	28.6	38.8	55.6	7.0	23.0	54.6	75.6	39.5	16.5	6.2	4.0	29.7	34.7
One-stage																	
YOLOV5-s^33^	CSPDarkNet	65.9	28.4	84.5	43.5	48.6	72.7	17.4	37.9	65.7	81.0	47.1	29.7	23.1	47.8	33.4	48.4
YOLOV5-m^33^	CSPDarkNet	63.8	34.5	85.4	45.0	52.2	71.6	18.7	32.4	66.6	81.8	49.0	27.2	27.0	44.1	33.3	48.8
YOLOV5-l^33^	CSPDarkNet	66.0	29.0	86.3	45.4	52.6	70.2	14.2	36.8	63.3	82.5	48.9	28.7	25.7	58.1	34.1	49.5
YOLOV5-x^33^	CSPDarkNet	63.8	25.5	87.8	43.8	54.5	70.3	18.4	34.9	64.9	83.5	49.3	28.0	22.7	53.4	31.5	48.8
PicoDet-s^42^	LCNet	57.9	36.7	84.6	42.0	48.1	75.1	14.0	33.6	61.6	80.0	43.4	24.0	12.8	54.9	31.7	46.7
PicoDet-m^42^	LCNet	59.4	26.8	83.0	43.1	48.6	78.8	16.2	32.2	62.8	78.3	42.1	22.4	20.0	45.5	28.8	45.9
PicoDet-l^42^	LCNet	64.1	28.7	83.3	41.4	47.1	77.4	11.2	35.2	63.5	79.5	44.0	22.8	13.9	45.5	30.6	45.9
PicoDet-xs^42^	LCNet	51.8	26.6	82.1	40.1	48.4	73.6	16.3	31.5	63.2	75.9	41.0	17.0	9.3	56.2	31.1	44.3
TOOD^43^	ResNet	31.7	28.9	82.4	40.1	47.0	57.2	20.1	35.3	60.8	74.7	38.7	17.6	15.6	56.6	25.7	42.2
PP-YOLOE -s^45^	CSPResNet	67.9	35.8	86.6	47.6	55.6	79.7	18.6	38.5	66	82	47.4	24.5	22.3	62.2	35.2	51.3
PP-YOLOE -m^45^	CSPResNet	69.0	38.8	84.5	49.4	54.6	81.0	19.2	38.0	66.4	81.7	48.0	25.5	22.0	66.6	35.1	52.0
PP-YOLOE -l^45^	CSPResNet	68.6	41.5	85.6	47.7	53.8	80.8	20.1	32.0	65.6	83.2	48.8	25.9	17.9	64.0	35.2	51.4
PP-YOLOE -x^45^	CSPResNet	63.9	41.4	86.7	51.4	58.1	78.7	23.4	37.3	66.5	82.7	49.8	26.4	19.0	60.5	33.9	52.0
RTMDet-s^44^	CSPNeXt	53.9	39.7	85.8	43.4	56.9	67.6	22.9	35.1	60.7	82.2	49.3	23.5	21.3	56.6	34.2	48.9
RTMDet-m^44^	CSPNeXt	52.9	30.3	82.6	42.0	53.5	68.1	20.3	28.5	59.8	80.8	47.4	25.5	16.0	55.1	32.6	46.3
RTMDet-l^44^	CSPNeXt	61.9	39.9	82.7	43.6	52.6	69.1	19.8	30.6	56.1	80.6	47.3	24.8	19.3	70.0	32.5	48.7
RTMDet-x^44^	CSPNeXt	64.8	42.7	82.7	45.8	56.0	66.7	20.1	38.6	62.7	82.7	47.3	26.8	23.0	63.4	33.4	50.4
YOLOX-s^39^	CSPDarkNet	46.5	26.6	79.1	31.0	41.9	69.4	11.6	23.9	48.9	73.8	42.9	17.1	11.7	50.0	31.9	40.4
YOLOX-m^39^	CSPDarkNet	48.4	28.9	82.1	36.3	44.5	70.2	13.4	26.7	51.3	77.6	44.8	20.2	13.3	51.1	30.8	42.6
YOLOX-l^39^	CSPDarkNet	49.9	32.1	81.9	39.4	48.8	66.5	17.8	29.9	52.9	80.8	44.6	25.2	17.5	50.1	32.6	44.7
YOLOX-x^39^	CSPDarkNet	50.9	33.3	83.6	41.7	49.7	64.0	18.6	36.7	58.8	81.1	49.1	25.4	20.3	53.4	34.6	46.7
YOLOv7^40^	ELAN	73.2	38.1	86.6	45.7	60.5	79.2	16.2	36.4	65.8	85.7	50.8	22.1	27.0	53.2	31.9	51.5
YOLOv7x^40^	ELAN	72.8	35.0	86.9	46.8	58.5	77.5	20.1	39.0	67.0	86.0	50.2	25.5	29.8	54.3	32.1	52.1
YOLOv7-w6^40^	ELAN	72.9	37.0	85.0	50.4	61.5	82.1	17.5	38.8	67.7	84.5	51.6	28.2	26.8	44.3	33.8	52.1
YOLOv7-e6^40^	ELAN	64.6	30.7	84.8	47.2	55.5	74.7	18.1	44.8	61.0	82.9	48.3	25.8	19.2	54.2	34.6	49.8
YOLOv7-d6^40^	ELAN	67.9	31.2	83.7	43.9	54.1	78.7	17.6	41.6	60.3	81.7	46.2	22.8	13.3	53.2	33.3	48.6
YOLOv7-e6e^40^	ELAN	66.5	29.2	83.3	42.5	50.3	77.7	14.6	38.6	58.5	79.6	45.1	26.3	22.6	48.7	32.2	47.7
YOLOv8-n^41^	CSPDarkNet	65.9	36.0	86.3	46.6	56.8	75.2	17.0	41.6	67.8	85.9	48.2	30.5	23.3	66.8	34.4	52.2
YOLOv8-s^41^	CSPDarkNet	71.2	37.5	87.6	44.6	57.5	77.6	18.4	37.3	70.7	85.2	49.9	31.1	31.9	55.5	33.9	52.7
YOLOv8-m^41^	CSPDarkNet	76.3	34.9	88.0	52.7	60.7	79.4	22.9	40.6	70.7	86.4	50.8	28.9	25.3	51.2	30.8	53.3
YOLOv8-l^41^	CSPDarkNet	72.7	35.3	89.3	49.7	59.9	76.8	15.9	38.4	68.8	85.0	50.6	28.9	29.0	57.0	31.6	52.6
YOLOv8-x^41^	CSPDarkNet	74.3	38.2	89.3	46.0	58.7	78.8	21.9	41.0	71.5	87.1	51.1	28.3	30.3	55.4	31.2	53.5
Proposed Method	FS	71.5	35.9	86.1	48.6	60.1	77.4	22.0	40.0	66.6	86.9	50.9	30.1	23.8	54.8	33.1	52.5
Proposed Method	FSS	75.2	40.1	87.8	52.9	61.9	83.0	23.1	44.7	69.1	87.1	52.3	30.7	30.2	60.2	34.1	55.5

As can be seen from Table 5, the mAP of misc, can, tire, plastic, rod, and metal are less than 0.5, on the one hand, because the training samples of the dataset are small (the number of training samples are 170, 89, 488, 136, 17, 30, respectively), on the other hand, the objects are deformed due to the water flow, image resolution, light refraction, etc. The object features learned by the model are not completely consistent with the inherent attribute features of the object. One of the lowest detection accuracies is metal, except for the reason of the small sample dataset, when labeling the dataset, the tin, coin, iron cage, rusty anchor, and tin can are marked as metal, even though the FSA networks model incorporates attention module, it can only extract the abstract features of the object, and the mAP of metal is only 23.1%. Due to the small number of sample sets in rod, the focal loss was used in the programming to solve the problem of lower accuracy due to sample imbalance, and the final mAP was only 30.2%. During the acquisition of the datasets, some of the plastic was in the marine soil, some was floating on the ocean surface, and some of the plastic overlapped with other object samples, making the features learned by the model incomplete, resulting in a final mAP of only 30.7%.

In summary, the FSS module and group SPPCSPC module introduced in the FSA networks can extract shallow features extraction, deep features, and reconstruct the image, while reducing the number of parameters; CCA focuses more attention on the dense object feature region, while introducing the residual operation to improve the fusion ability of shallow and deep feature maps; the joint use of the auxiliary head and the lead head at the output end allows the lead head to focus on learning the remaining features that have not yet been learned, effectively improving the feature extraction ability of objects in complex environments and making the model more advantageous when dealing with complex submarine garbage image object detection tasks.

### Visualization analysis

**Figure 2:**
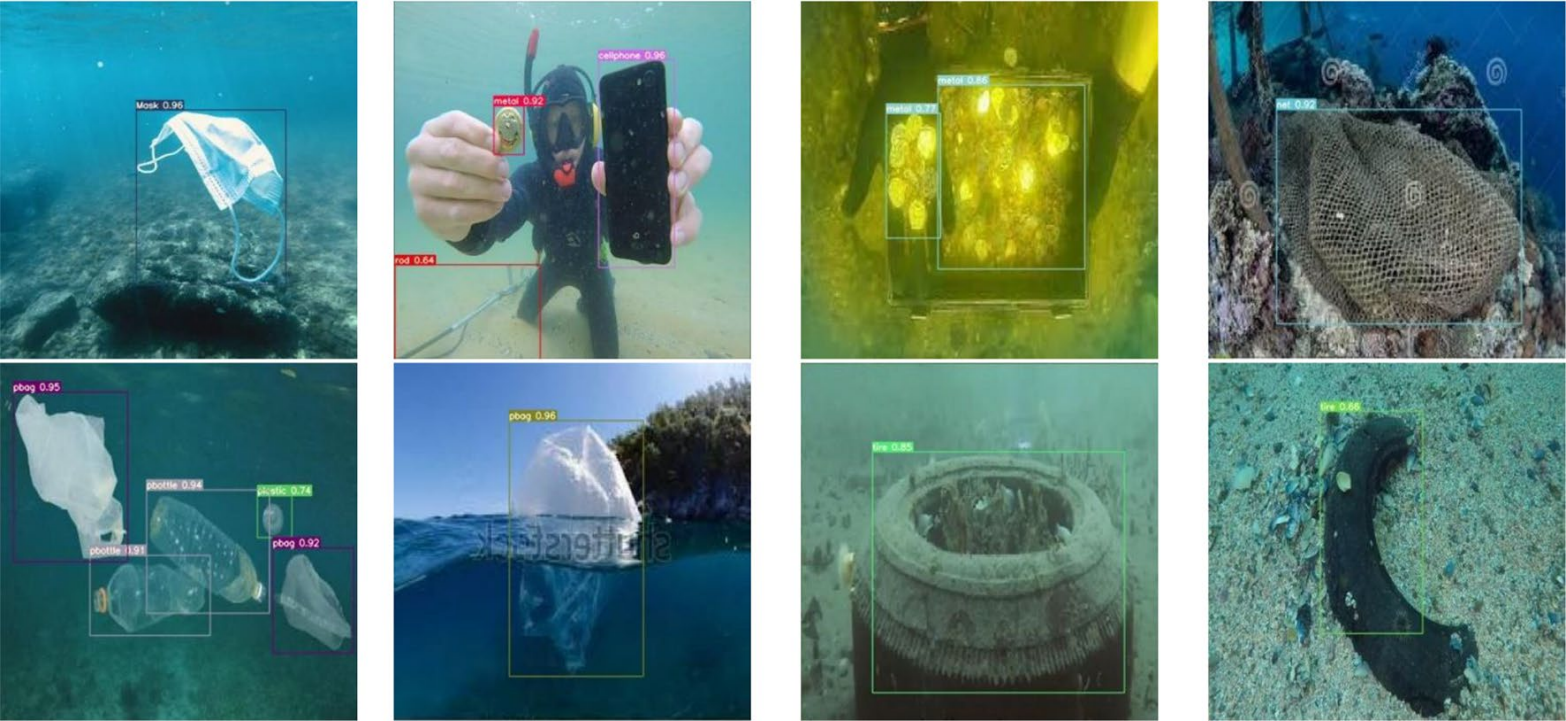
Object detection results.

This paper uses representative and difficult images from the submarine garbage test set to evaluate the actual results of the algorithm for all classes of objects and visualize and analyze them. The detection results are shown in Fig. 2. As can be seen from the figures, the object detection accuracy is generally high, and even if there is an occlusion or object at the junction of water bodies, it can be detected very well.

**Figure 3:**
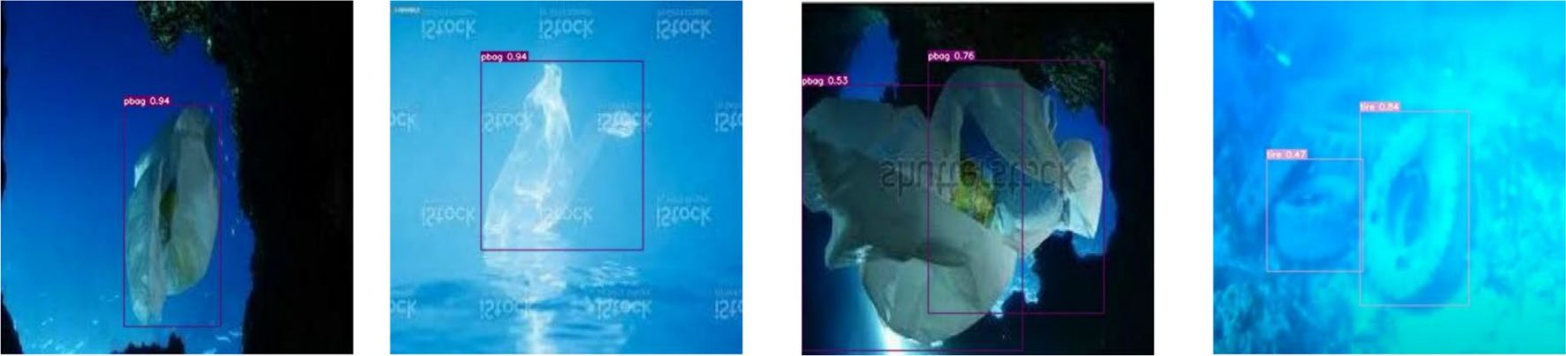
Detection results under illumination changes.

**Figure 4:**
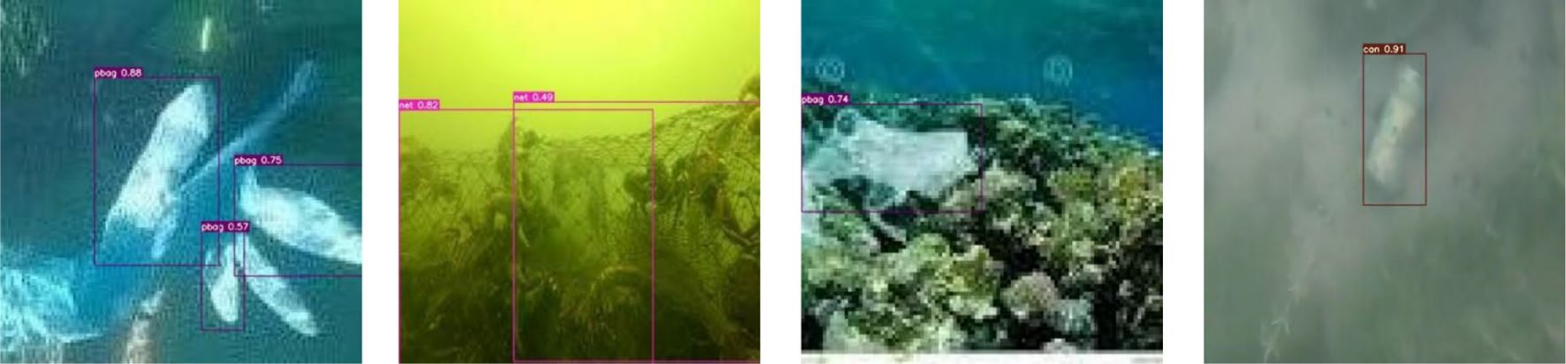
Fuzzy image detection results.

**Figure 5:**
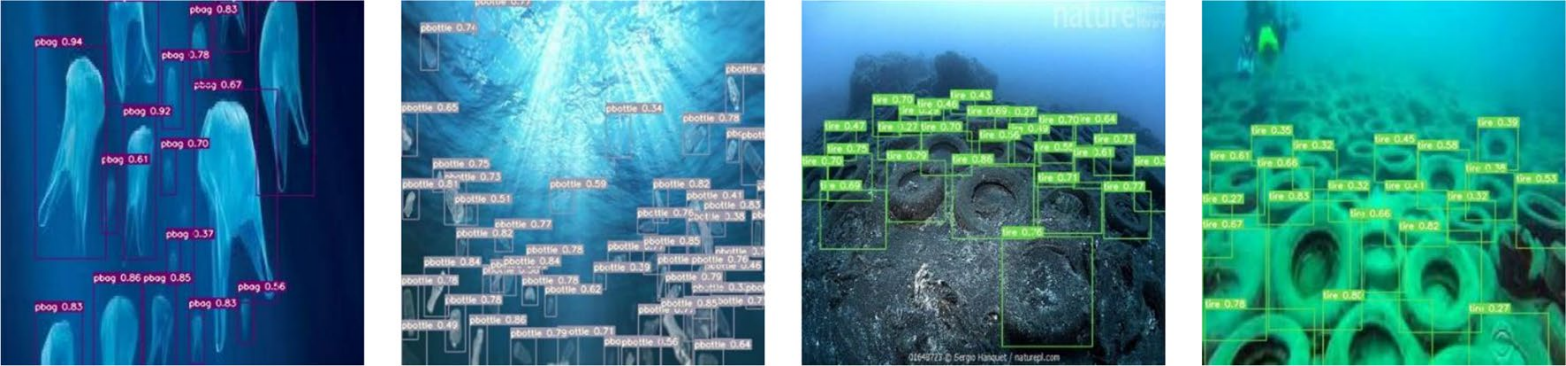
Small object detection results.

**Figure 6:**
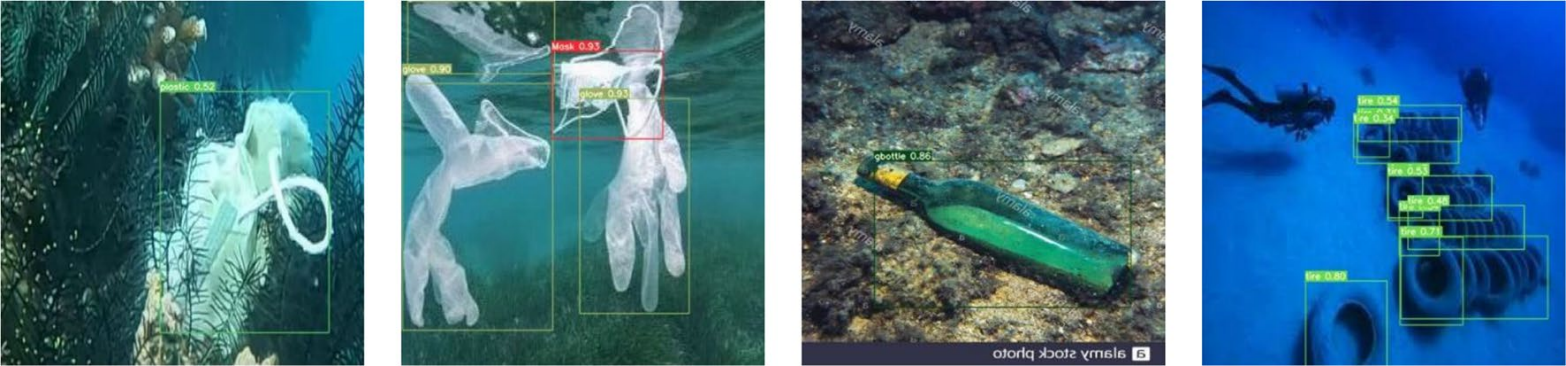
Object under occlusion detection results.

Figure 3 shows the detection effect in the case of significant light variation. There exists dim scene in the figures, and the object color is similar to the background color, the results illustrate that the model is less affected by the light variation and has better detection ability. Figure 4 presents images of fuzzy distortion caused by underwater shooting, and the detection results reveal that the model can detect the objects in the fuzzy scene, which indicates that the model has good robustness. Figure 5 represents the detection effect of dense small objects, where the pbag, pbottle, and tire are extremely small, however, all of them can be detected precisely, which illustrates the model’s outstanding detection ability for small objects as well. The detection effect in the presence of occlusion is shown in Fig. 6. The results demonstrate that the FSA network model is able to detect the object correctly even in the presence of occlusion by other objects, or incomplete objects.

**Figure 7:**
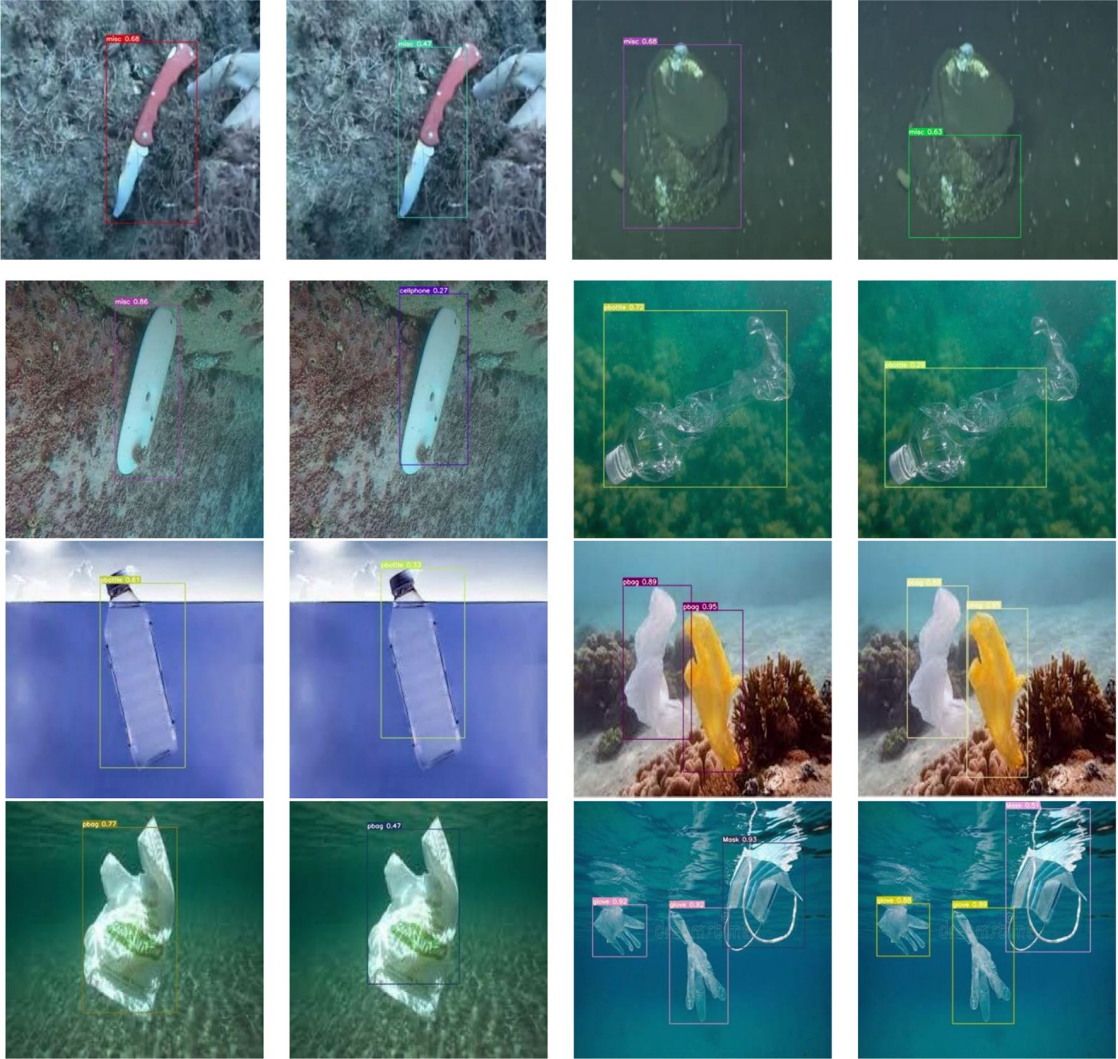
Detection results (boundary box).

**Figure 8:**
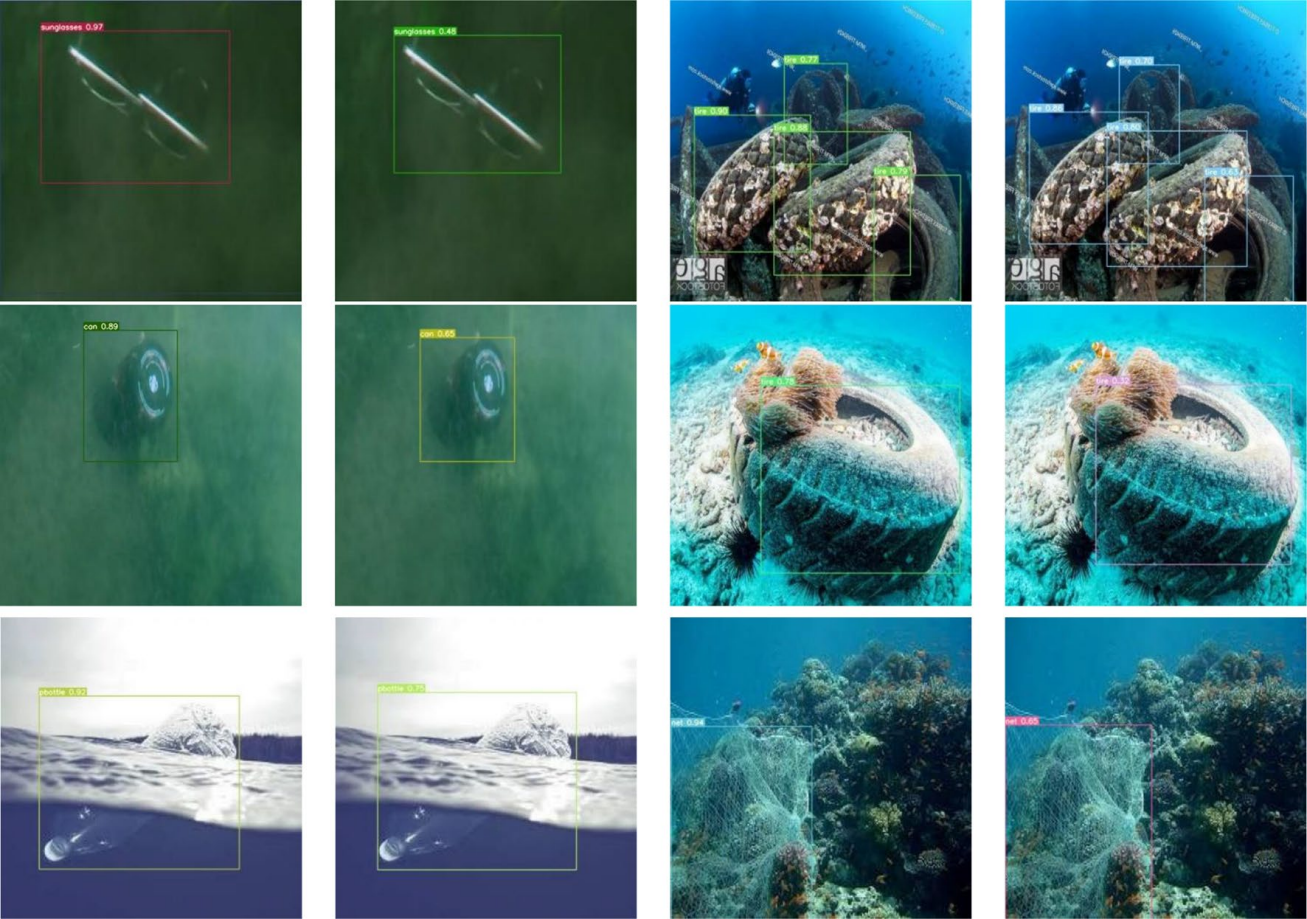
Detection results (accuracy).

Pictures in the left column of Figs. 7 and 8 are the FSA detection results, and the right column are the YOLOv7 detection results. As can be seen from Fig. 7, the FSA detection results show more accurate bounding box, while YOLOv7 has a situation where the bounding box is too large or too small. Consequently, it proves that the prediction box obtained by CIoU used in this paper is more consistent with the real position of the object.

Figure 8 demonstrates that the detection results of the FSA networks model are more accurate and have better detection effects. Therefore, it can be demonstrated that the FSA networks model, through the attention module, increases the perceptual field of the feature map, strengthens the feature extraction ability of the network for small objects, and can reserve more feature information of the object area. The combination of auxiliary head and lead head weakens the interference of background noise, fuses shallow and deep features, improves the global feature extraction ability, and has better performance in dealing with object detection in complex backgrounds, which not only reduces missed and false detections, but also is less affected by environmental and illumination changes. Consequently, the FSA networks model has higher detection accuracy and more precise detection results. Overall, the FSA networks model can obtain more accurate object positions, has better robustness to illumination changes, and the object detection effect is obviously improved in sophisticated backgrounds, and the inference speed can reach 72.15 FPS.

## Discussion

In this paper, we propose a one-stage detector, full stage auxiliary networks with auxiliary focal loss and multi-attention module, based on YOLO. It aims to improve the performance of dense small object detection in complex backgrounds for real-time submarine garbage object detection tasks. In order to avoid overfitting and improve the generalization ability of the model, data augmentation is performed using left–right inversion, mosaic, mix up and other strategies for the submarine garbage dataset. Then, utilizing channel streaming, cross-stage connection strategy to obtain all features of each stage and hierarchical cross features, the criss-cross attention module added afterwards better extracts the deep abstract features of the full stage by calculating the distance of intra-class and inter-class features, which makes the obtained features more focused on the intensive features of small objects. In the regression analysis stage, the auxiliary focal loss function is used to calculate the object class and confidence level to balance the problem of unbalanced positive and negative samples, focus the training on difficult samples, and improve the overall detection accuracy. The experimental results demonstrate that the FSA networks achieved state-of-the-art performance compared with the mainstream networks, while ensuring high efficiency in inference, and can be applied to real-time object detection tasks.

Although, the FSA networks proposed in this paper is excellent in terms of performance and accuracy, the number of parameters and computational effort are greatly increased by introducing the attention module at the end of each FSS module, and the effect of model width is not discussed. Therefore, it can be further investigated how to reduce the computational overhead brought by the addition of the attention module while increasing the model width.

## Methods

### Overall architecture

**Figure 9:**
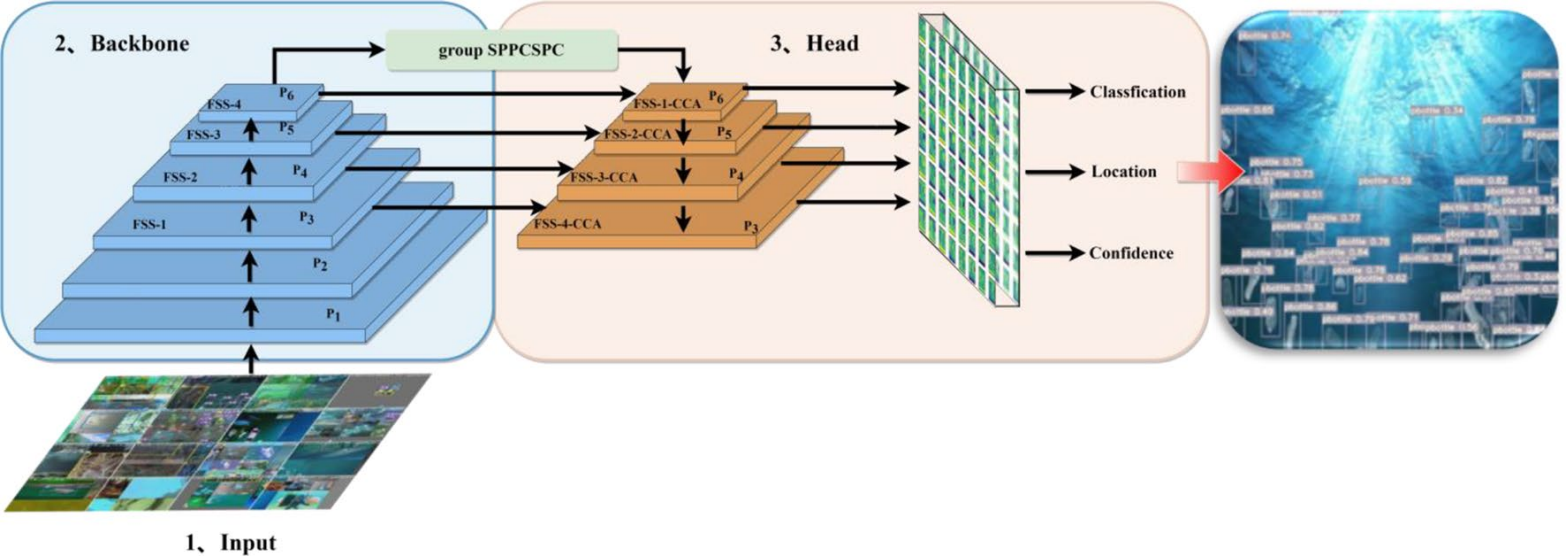
The structure of full stage auxiliary networks.

The detector proposed in this paper called full stage auxiliary networks (FSA Networks) is based on the YOLO detection framework. As shown in Fig. 9, the images are performed by data enhancement before being sent to the backbone. P*i* (*i* ∈ [1–6]) indicates that the feature map image size output by the current layer is 1/2^*i*^ of the original image, and after the group SPPCSPC convolution operation, the size of the feature map of the input head is 1/64 of the original image. FSS-*i* indicates the full stage shortcut convolution operation for the current layer respectively. In backbone and head, FSS is used to extract shallow features and deep features respectively, and the operation of attention mechanism is added behind the FSS in head to obtain image context information from each pixel vertical and horizontal path, so that the model can focus more on capturing feature information of dense small target regions and reduce the noise interference of complex background. The outputs of P3, P4, P5 and P6 in FSS in backbone are used as auxiliary heads in the regression analysis of classification, location and confidence, and the loss is calculated together with the lead head of each layer output of FSS-CCA.

### Full stage convolution

**Figure 10:**
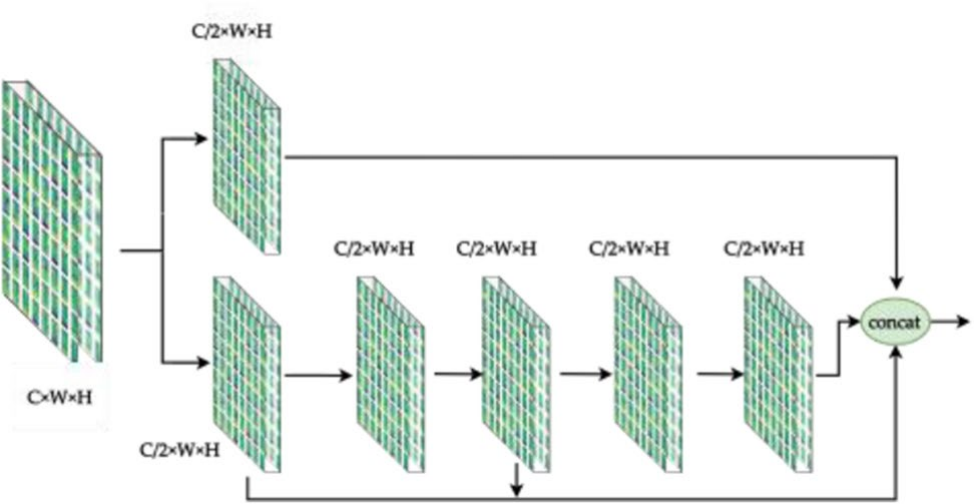
Half stage convolution module.

**Figure 11:**
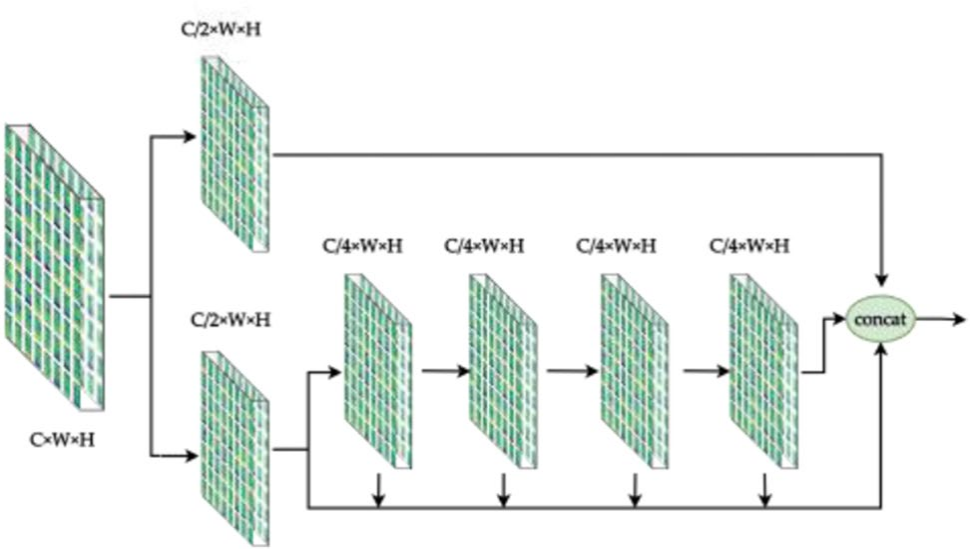
Full stage convolution module.

Different from the backbone of CSPNet and YOLOv7, The HS (Fig. 10) and FS (Fig. 11) modules preserve DenseNet's advantage of reusing features, while preventing excessive repetitive gradient information transfer and learning by truncating the gradient flow, mainly through a hierarchical feature fusion strategy. First, split the upper feature map into two parts, one part goes through the stage and transition layers, and the other part concatenates with the transmitted feature map to the next stage. The module implementation extends the number of channels and bases of the computational module by group convolution, uses channel streaming, and cross-stage connection strategy, retains all features of the upper layer, fuses the channel features of each stage, and finally, the output channel is twice as many as the input channel, which can acquire more features in depth and width at the same time, better preserves the actual feature structure of the object, and makes the model more robust and has stronger generalization ability.

**Figure 12:**
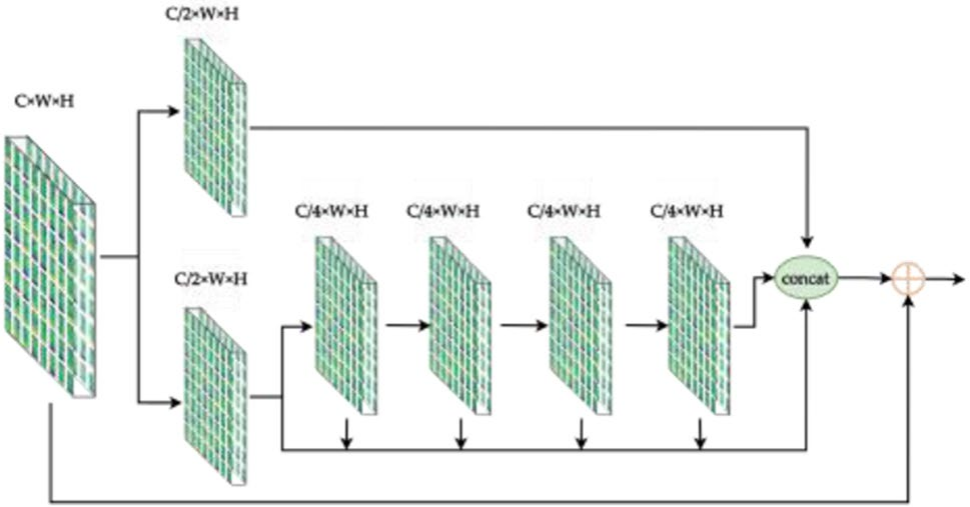
Full stage shortcut convolution module.

Figure 9 demonstrates that in order to obtain feature maps at various scales, each FS module in the head must be upsampled. However, this adds irregular pixels, which causes the image to lose some of its finer details. In order to better reuse the features in the backbone and compensate for the missing data introduced by upsampling, the FS-*i* feature map in the backbone is transmitted to the FS-*i* layer corresponding to P_*i*_ in the head in this study. The shortcut is used to connect the residuals with the features at the end of the FS module, thereby employing both the full features before and after upsampling. Therefore, the FSS (Full Stage Shortcut) module (Fig. 12) is used for all the backbone and head in this paper.

### Attention module

In recent years, the attention model has been widely used in image processing^46^, speech recognition^47^, natural language processing^48^, and other fields^49^. The quality of attention module is a set of weight coefficients that are learned independently through the network, and it emphasizes the areas of our interest while suppressing irrelevant background areas in a “dynamic weighting” way.

Therefore, in order to reduce the GPU occupancy, use larger batch size and improve the detection accuracy, this paper uses the CCA^50^(Criss-Cross Attention) module to upgrade the model. Given a local feature maps ${\text{F}}{  \in  }{\text{R}}^{\text{C}}\times{\text{W}}\times{\text{H}}$, two feature maps G and K are generated by two 1 × 1 convolutions layers respectively, where $\text{G, K}{  \in  }{\text{R}}^{{{\text{C}}^{\prime}}}{\times{\text{W}}\times {\text{H}}}$, C’ is the number of channels, which is less than C for dimension reduction. After obtaining feature maps G and K, the three-dimensional feature map with the shape of C’ × H × W can be easily reshaped into a two-dimensional C’ × (H × W) matrix. The attention map A ${  \in  }{\text{R}}^{{\text{(H}}+{\text{W}}-\text{1})\times {\text{W}}\times{\text{H}}}$ is generated by the Affinity operation. For each position u in the feature map G, a vector ${\text{G}}{\in}{\text{R}}^{{C^{\prime}}}$ with dimension C’ can be obtained. At the same time, the set $\Omega  \text{u}{\in}{\text{R}}^{{\text{(W}}+{\text{H}}-\text{1})\times C^{\prime}}$ can also be obtained from the feature map K which belongs to the same row or column with position u.

**Figure 13:**
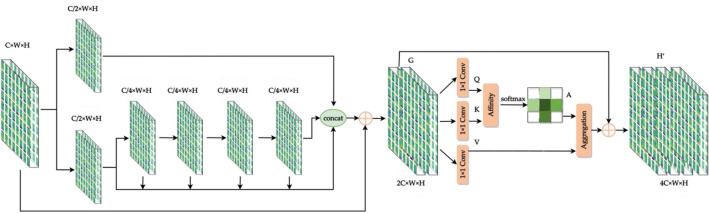
Full stage shortcut criss-cross attention module.

The features acquired by the FSS module are hierarchical cross features, therefore, adding the CCA module after the FSS module (Fig. 13) can obtain the category consistency loss and better extract more in-depth abstract features by calculating the distance between intra-class and inter-class features while preserving the feature structure.

### Auxiliary focal loss function

The one-stage method discards the stage of generating candidate boxes in order to improve the detection speed, and directly classifies the anchor boxes at a fine-grained level, so many boxes are predicted, but few boxes contain the correct object, leading to the category imbalance problem. In order to solve this problem, Lin^26^ proposed the focal loss function on the basis of the two-category balanced cross-entropy loss function, adding a weight factor in front of each category to solve the problem of unbalanced positive and negative samples, and adjusting the factor (γ ≥ 0 is an adjustable focusing parameter) to reduce the weight of easy-to-classify samples, focus on the training of difficult samples, and prevent easy-to-classify samples from dominating the gradient transfer. The definition is as follows:

1 $${F}_{L}=\left\{\begin{array}{l}-\alpha {\left(1-p\right)}^{r}\mathrm{log}(p)\\ -\left(1-\alpha \right){p}^{r}\mathrm{log}\left(1-p\right) \end{array}\right.\begin{array}{l}if y=1\\ if y=0\end{array}$$

**Figure 14:**
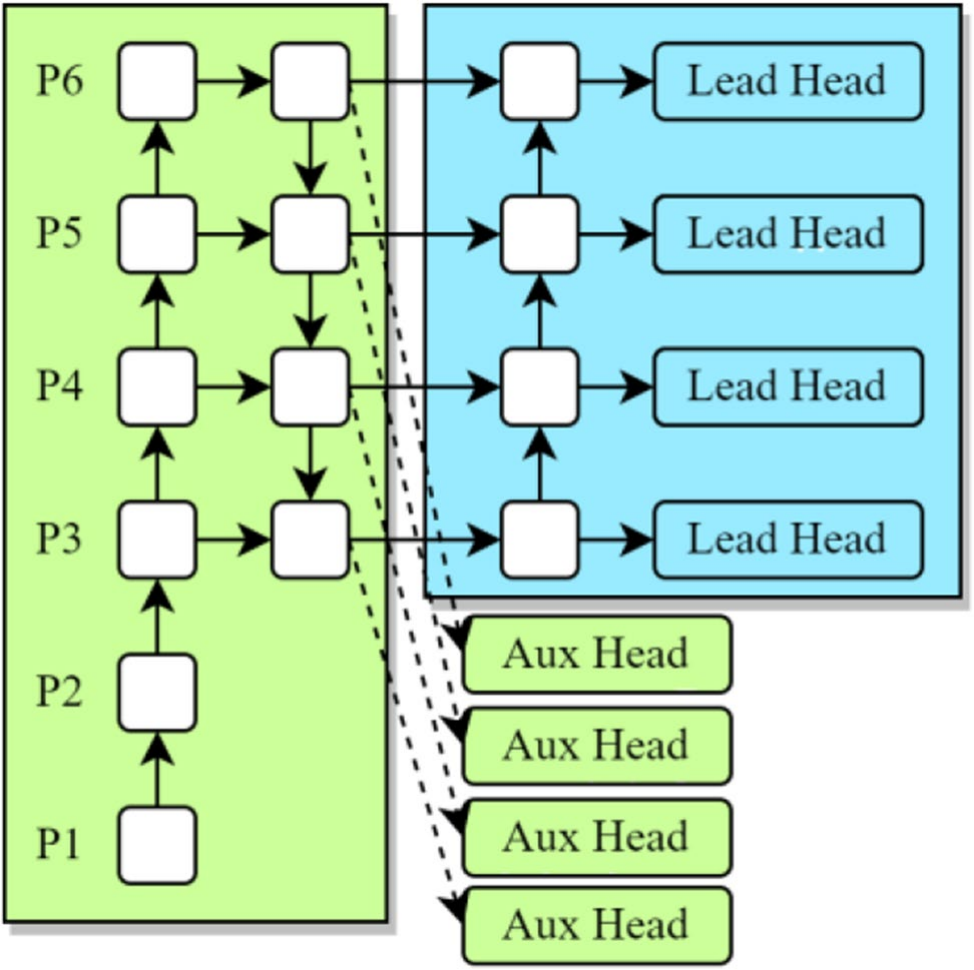
Lead head and auxiliary head.

**Figure 15:**
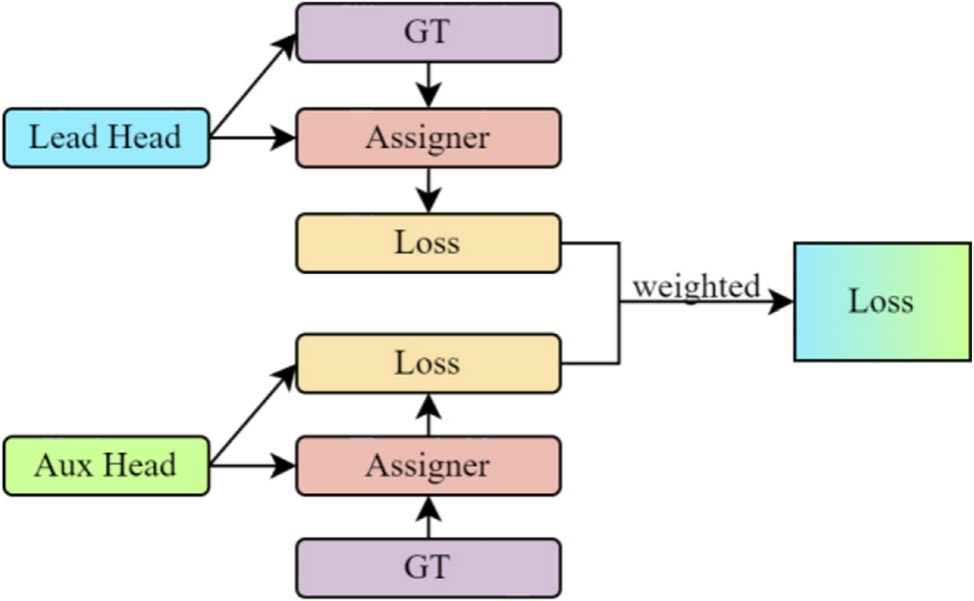
Loss with weighted different head.

In order to improve the overall accuracy and performance of the model, this paper uses the FSS module in the backbone to generate the auxiliary head for auxiliary training. The lead head generated by the FSS-CCA module is the main prediction result. Different from YOLOv7, the lead head and auxiliary head (Fig. 14) participate in the optimization model simultaneously and assign different weights (Fig. 15) to calculate classification, confidence, and regression losses. This is done to reduce the impact of the auxiliary head's "coarse" label and prevent a reduction in the lead head's detection accuracy. The lead head and auxiliary head both extract the IoU of the top 20 samples for summing in the actual calculation, and the classification and regression loss weights are set to 1:0.25. Similar to YOLOv5, the confidence loss is set at a ratio of 1/4 based on the output scale of the detection head. According to Fig. 9, the output contains 4 scales (1/8, 1/16, 1/32, 1/64), so it is very suitable for small object detection in multi-scale complex backgrounds.

Because of the additional training of the auxiliary head, the focal loss functions all need to be added for synchronous training, and the modified auxiliary focal loss function is as follows:

2 $${F}_{L\_class}=b\cdot \left({F}_{L\_class\_lead}+\frac{1}{4}{F}_{L\_class\_aux}\right)$$

3 $${F}_{L\_conf}=b\cdot \sum_{i=0}^{l-1}\left[b(i){F}_{L\_conf\_lead}+{\frac{b(i)}{4}F}_{L\_conf\_aux}\right]$$

where *b* is the batch size during training,* l* is the number of groups of detection heads (in this paper, there are 4 groups of auxiliary head and lead head, therefore, *l* = 4), *b*(*i*) = [4, 1, 1/4, 1/16] is the balance factor of auxiliary head and lead head.

### Materials: dataset and health check

To validate the robustness and generalization property of the proposed model, an opensource submarine garbage dataset is used to learn all types of labeled objects without undergoing any human screening, and the ratio of training sets validation sets, and test sets is set to 0.7:0.2:0.1 (Table 6). The dataset consists of 5,136 images of marine debris in 15 categories, with 2.3 labels per image. The aspect ratio distribution for each classification of dimension insight are shown in Table 7, most of the aspect ratios are images with median width multiplied by median height (300 × 199 pixel), and a few categories, such as cellphone, have high aspect ratios.

**Table 6:** Submarine garbage datasets.

Class name	Labels per image	Label numbers	Image numbers	Image numbers (Train70%)	Image numbers (Validation20%)	Image numbers (Test10%)
Tire	4.6	3223	702	488	145	69
pbag (plastic bag)	1.2	1632	1389	970	291	128
Mask	2.8	1574	568	456	79	33
pbottle (plastic bottle)	2.0	1342	663	479	122	62
Glove	2.6	1263	483	422	38	23
Net	1.0	745	710	500	147	63
gbottle (glass bottle)	2.2	484	223	160	36	27
Cellphone	1.1	385	335	234	61	40
Plastic	1.3	275	218	136	51	31
Misc	1.0	257	248	170	48	30
Electronics	1.4	196	138	97	27	14
Can	1.4	163	120	89	18	13
Metal	2.0	83	42	30	10	2
Rod	1.5	37	25	17	7	1
Sunglasses	1.0	18	18	14	3	1
All	2.3 (average)	11677	5136	3628	1007	501

**Table 7:** Aspect ratio distribution.

Aspect ratio	Number of images	Ratio
Tall	343	< 1:1
Square	208	1:1
Wide	4543	1:1.5
Very wide	37	> 1.5:1
Extremely wide	5	> 2.5:1

It can be seen from the distribution of the original dataset that there is a serious sample imbalance in the labeled images. So, some strategies need to be taken to expand the dataset, such as Random Erasing Data Augmentation^51^, RandAugment^52^, Mixup^53^,Cutout^54^, CutMix^55^, Mosaic^32^, Copy-Paste^56^, etc.

**Figure 16:**
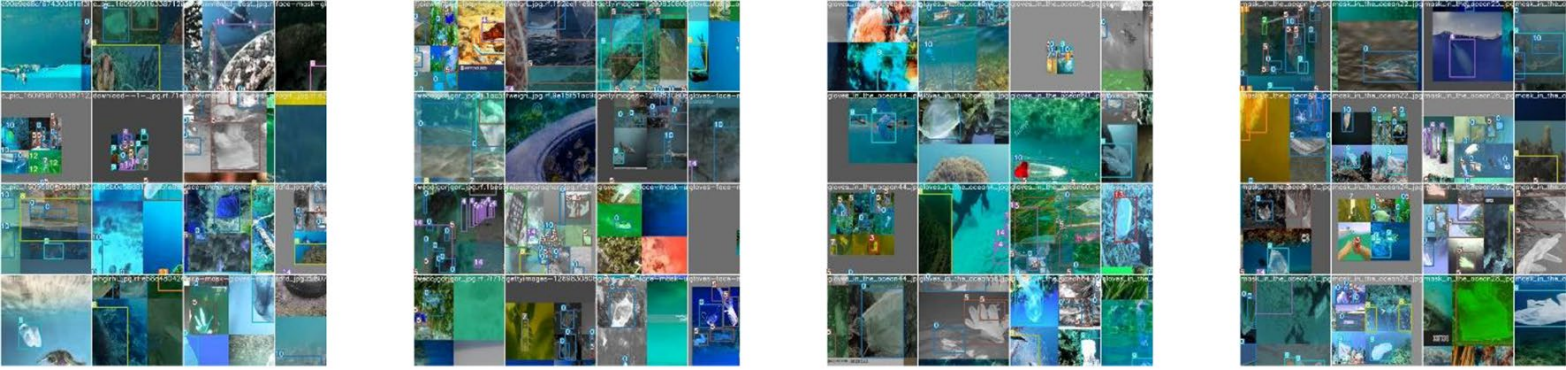
Images after data enhancement.

In this paper, we adjust hue, saturation, and value in the HSV color model, and enhance it by rotating 10 degrees and shifting the range to [−0.2, 0.2]. At the same time, we expand the dataset utilizing left–right inversion, Mosaic, Mix-Up, and Copy-Paste. Images after data enhanced shown in Fig. 16.

## Data Availability

The datasets analyzed during the current study is available at https://universe.roboflow.com/ncwu-mdh99/submarine-garbage.
